# Methyl 2-methyl-3,5-dinitro­benzoate

**DOI:** 10.1107/S1600536809055494

**Published:** 2010-01-09

**Authors:** Abdul Rauf Raza, Aisha Saddiqa, M. Nawaz Tahir, Muhammad Danish, Mohammad Saeed Iqbal

**Affiliations:** a Department of Chemistry, University of Sargodha, Sargodha, Pakistan; b Department of Physics, University of Sargodha, Sargodha, Pakistan; c Department of Chemistry, Government College University, Lahore, Pakistan

## Abstract

In the title compound, C_9_H_8_N_2_O_6_, the methyl ester group is almost planar (r.m.s. deviation = 0.002 Å) and is oriented at a dihedral angle of 24.27 (16)° with respect to the benzene ring. The nitro groups make dihedral angles of 4.2 (5)° and 60.21 (11)° with the benzene ring. In the crystal, mol­ecules are linked by C—H⋯O inter­actions, resulting in zigzag chains.

## Related literature

For a related structure, see: Tahir *et al.* (2009 ▶). 
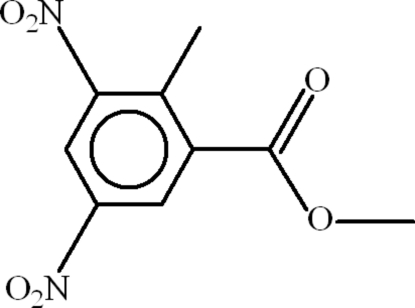


## Experimental

### 

#### Crystal data

C_9_H_8_N_2_O_6_
*M* *_r_* = 240.17
Orthorhombic, 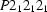

*a* = 6.7948 (5) Å
*b* = 8.8478 (8) Å
*c* = 17.3539 (17) Å
*V* = 1043.30 (16) Å^3^
*Z* = 4
Mo *K*α radiation
μ = 0.13 mm^−1^
*T* = 296 K
0.30 × 0.10 × 0.09 mm

#### Data collection

Bruker Kappa APEXII CCD diffractometer
Absorption correction: multi-scan (*SADABS*; Bruker, 2005 ▶) *T*_min_ = 0.985, *T*_max_ = 0.987
6284 measured reflections
1510 independent reflections
1001 reflections with *I* > 2σ(*I*)
*R*_int_ = 0.034

#### Refinement

*R*[*F* ^2^ > 2σ(*F* ^2^)] = 0.041
*wR*(*F* ^2^) = 0.097
*S* = 1.02
1510 reflections
156 parameters
H-atom parameters constrained
Δρ_max_ = 0.16 e Å^−3^
Δρ_min_ = −0.17 e Å^−3^

### 

Data collection: *APEX2* (Bruker, 2007 ▶); cell refinement: *SAINT* (Bruker, 2007 ▶); data reduction: *SAINT*; program(s) used to solve structure: *SHELXS97* (Sheldrick, 2008 ▶); program(s) used to refine structure: *SHELXL97* (Sheldrick, 2008 ▶); molecular graphics: *ORTEP-3* (Farrugia, 1997 ▶) and *PLATON* (Spek, 2009 ▶); software used to prepare material for publication: *WinGX* (Farrugia, 1999 ▶) and *PLATON*.

## Supplementary Material

Crystal structure: contains datablocks global, I. DOI: 10.1107/S1600536809055494/hb5296sup1.cif

Structure factors: contains datablocks I. DOI: 10.1107/S1600536809055494/hb5296Isup2.hkl

Additional supplementary materials:  crystallographic information; 3D view; checkCIF report

## Figures and Tables

**Table 1:** Hydrogen-bond geometry (Å, °)

*D*—H⋯*A*	*D*—H	H⋯*A*	*D*⋯*A*	*D*—H⋯*A*
C9—H9*C*⋯O2^i^	0.96	2.56	3.353 (4)	140

Symmetry code: (i) 
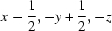
.

**Fig. 1.**
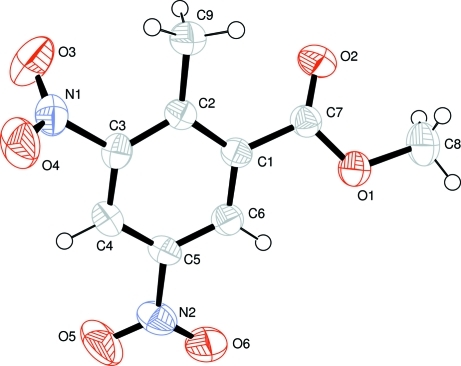
View of (I) with displacement ellipsoids drawn at the 50% probability level.

**Fig. 2.**
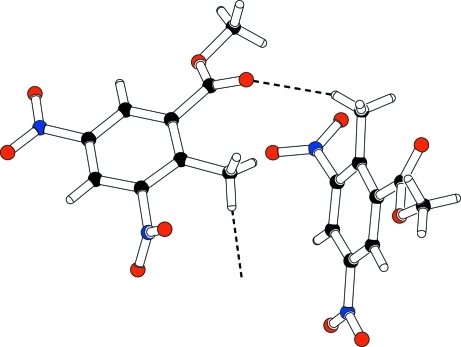
Partial packing diagram of (I).

## supplementary crystallographic information

### Comment

Our work is aimed at the synthesis of various isocoumarins and the title compound (I, Fig. 1) is an intermediate for their preparation.

We have reported crystal structures of 2-Methyl-3,5-dinitrobenzoic acid (Tahir *et al.*, 2009) and the title compound is its methyl ester.

In the title compound benzene ring A (C1–C6) is of course planar. The methyl ester B (O2/C7/O1/C8) is also planar with a maximum r. m. s. deviation of 0.0014 Å from the mean square plane. The dihedral angle between A/B is 24.27 (16)°. Two nitro groups C (O3/N1/O4) and D (O5/N2/O6) are oriented at dihedral angles of 60.21 (11)° and 4.22 (51)° respectively, with the benzene ring. The dihedral angle between C/D is 63.24 (25)°. The molecules are stabilized due to intra as well inter-molecular and C–H···O interactions (Table 1, Fig. 2) in the form of zigzag polymeric chains.

### Experimental

H_2_SO_4_ (5 ml) was added as a catalyst to a stirred solution of 2-methyl-3,5-dinitrobenzoic acid (1 g, 4.4 mmol) (Tahir *et al.*, 2009) in MeOH (20 ml) and refluxed for 5 h. The progress of reaction was monitored by TLC. The crystals were immediately obtained upon gradual cooling followed by pouring reaction mixture to beaker. The crude product was filtered and consecutive washing with MeOH and H_2_O afforded impure crystals of (I). The recrystalization from CHCl_3_ afforded (69.3%) colourless needles of the title compound (I).

### Refinement

In the absence of significant anomalous scattering effects, Friedal pairs were merged. The H-atoms were positioned geometrically (C—H = 0.93–0.96 Å) and refined as riding with *U*_iso_(H) = *xU*_eq_(C), where *x* = 1.2 for aryl and 1.5 for methyl H atoms.

### Crystal data

**Table:**

C_9_H_8_N_2_O_6_	*F*(000) = 496
*M**_r_* = 240.17	*D*_x_ = 1.529 Mg m^−^^3^
Orthorhombic, *P*2_1_2_1_2_1_	Mo *K*α radiation, λ = 0.71073 Å
Hall symbol: P 2ac 2ab	Cell parameters from 1510 reflections
*a* = 6.7948 (5) Å	θ = 2.4–28.3°
*b* = 8.8478 (8) Å	µ = 0.13 mm^−^^1^
*c* = 17.3539 (17) Å	*T* = 296 K
*V* = 1043.30 (16) Å^3^	Needle, colourless
*Z* = 4	0.30 × 0.10 × 0.09 mm

### Data collection

**Table:**

Bruker Kappa APEXII CCD diffractometer	1510 independent reflections
Radiation source: fine-focus sealed tube	1001 reflections with *I* > 2σ(*I*)
graphite	*R*_int_ = 0.034
Detector resolution: 7.40 pixels mm^-1^	θ_max_ = 28.3°, θ_min_ = 2.4°
ω scans	*h* = −5→9
Absorption correction: multi-scan (*SADABS*; Bruker, 2005)	*k* = −11→9
*T*_min_ = 0.985, *T*_max_ = 0.987	*l* = −23→21
6284 measured reflections	

### Refinement

**Table:**

Refinement on *F*^2^	Primary atom site location: structure-invariant direct methods
Least-squares matrix: full	Secondary atom site location: difference Fourier map
*R*[*F*^2^ > 2σ(*F*^2^)] = 0.041	Hydrogen site location: inferred from neighbouring sites
*wR*(*F*^2^) = 0.097	H-atom parameters constrained
*S* = 1.02	*w* = 1/[σ^2^(*F*_o_^2^) + (0.0444*P*)^2^ + 0.0609*P*] where *P* = (*F*_o_^2^ + 2*F*_c_^2^)/3
1510 reflections	(Δ/σ)_max_ < 0.001
156 parameters	Δρ_max_ = 0.16 e Å^−^^3^
0 restraints	Δρ_min_ = −0.16 e Å^−^^3^

### Special details

**Table:**

Geometry. Bond distances, angles *etc*. have been calculated using the rounded fractional coordinates. All su's are estimated from the variances of the (full) variance-covariance matrix. The cell e.s.d.'s are taken into account in the estimation of distances, angles and torsion angles
Refinement. Refinement of *F*^2^ against ALL reflections. The weighted *R*-factor *wR* and goodness of fit *S* are based on *F*^2^, conventional *R*-factors *R* are based on *F*, with *F* set to zero for negative *F*^2^. The threshold expression of *F*^2^ > σ(*F*^2^) is used only for calculating *R*-factors(gt) *etc*. and is not relevant to the choice of reflections for refinement. *R*-factors based on *F*^2^ are statistically about twice as large as those based on *F*, and *R*- factors based on ALL data will be even larger.

### Fractional atomic coordinates and isotropic or equivalent isotropic displacement parameters (Å^2^)

**Table:**

	*x*	*y*	*z*	*U*_iso_*/*U*_eq_	
O1	0.7519 (3)	0.6592 (2)	0.08307 (14)	0.0613 (8)	
O2	0.9455 (3)	0.4581 (2)	0.07824 (14)	0.0640 (8)	
O3	0.4935 (4)	−0.0440 (3)	0.14759 (15)	0.0822 (10)	
O4	0.2279 (3)	0.0187 (2)	0.08784 (14)	0.0704 (9)	
O5	0.0268 (4)	0.4613 (3)	0.26197 (17)	0.0929 (10)	
O6	0.1892 (3)	0.6626 (3)	0.23914 (14)	0.0731 (9)	
N1	0.3765 (4)	0.0489 (3)	0.12346 (13)	0.0495 (8)	
N2	0.1635 (3)	0.5275 (3)	0.23353 (14)	0.0552 (10)	
C1	0.6122 (3)	0.4298 (3)	0.11981 (14)	0.0356 (8)	
C2	0.5898 (3)	0.2738 (3)	0.10750 (14)	0.0348 (8)	
C3	0.4175 (4)	0.2112 (3)	0.13733 (14)	0.0388 (8)	
C4	0.2759 (4)	0.2888 (3)	0.17786 (15)	0.0417 (9)	
C5	0.3090 (4)	0.4397 (3)	0.18916 (15)	0.0393 (8)	
C6	0.4714 (4)	0.5114 (3)	0.16018 (15)	0.0414 (9)	
C7	0.7890 (4)	0.5130 (3)	0.09141 (16)	0.0403 (9)	
C8	0.9151 (5)	0.7524 (3)	0.0570 (3)	0.0850 (16)	
C9	0.7325 (4)	0.1820 (3)	0.06173 (17)	0.0491 (10)	
H4	0.16360	0.24127	0.19659	0.0500*	
H6	0.48749	0.61477	0.16753	0.0497*	
H8A	1.02426	0.74060	0.09162	0.1274*	
H8B	0.87514	0.85644	0.05607	0.1274*	
H8C	0.95366	0.72162	0.00616	0.1274*	
H9A	0.83335	0.14382	0.09519	0.0737*	
H9B	0.79104	0.24423	0.02263	0.0737*	
H9C	0.66471	0.09904	0.03793	0.0737*	

### Atomic displacement parameters (Å^2^)

**Table:**

	*U*^11^	*U*^22^	*U*^33^	*U*^12^	*U*^13^	*U*^23^
O1	0.0486 (11)	0.0371 (11)	0.0983 (17)	−0.0068 (9)	0.0159 (12)	0.0010 (11)
O2	0.0356 (10)	0.0614 (13)	0.0950 (17)	0.0043 (10)	0.0103 (11)	0.0042 (13)
O3	0.104 (2)	0.0439 (13)	0.0988 (19)	0.0038 (14)	−0.0224 (16)	0.0074 (12)
O4	0.0598 (13)	0.0670 (14)	0.0843 (17)	−0.0270 (11)	−0.0044 (13)	−0.0147 (13)
O5	0.0706 (16)	0.0951 (18)	0.113 (2)	−0.0172 (15)	0.0572 (16)	−0.0307 (16)
O6	0.0693 (15)	0.0584 (15)	0.0916 (19)	0.0121 (12)	0.0221 (14)	−0.0147 (13)
N1	0.0574 (15)	0.0460 (15)	0.0450 (14)	−0.0120 (13)	0.0060 (12)	−0.0029 (11)
N2	0.0467 (14)	0.0669 (19)	0.0520 (16)	0.0031 (13)	0.0099 (12)	−0.0151 (14)
C1	0.0305 (12)	0.0395 (16)	0.0367 (14)	−0.0004 (11)	−0.0031 (11)	−0.0010 (11)
C2	0.0325 (13)	0.0404 (15)	0.0315 (13)	0.0011 (12)	−0.0033 (11)	0.0000 (11)
C3	0.0438 (15)	0.0372 (15)	0.0355 (14)	−0.0059 (13)	−0.0059 (12)	0.0005 (11)
C4	0.0354 (14)	0.0507 (17)	0.0389 (16)	−0.0069 (13)	0.0014 (12)	0.0003 (13)
C5	0.0311 (13)	0.0474 (16)	0.0395 (15)	0.0006 (12)	0.0022 (10)	−0.0071 (12)
C6	0.0380 (14)	0.0405 (15)	0.0457 (16)	−0.0013 (12)	−0.0032 (12)	−0.0018 (12)
C7	0.0357 (14)	0.0414 (16)	0.0437 (16)	−0.0032 (12)	−0.0005 (12)	−0.0011 (12)
C8	0.066 (2)	0.049 (2)	0.140 (4)	−0.0214 (17)	0.028 (2)	0.010 (2)
C9	0.0468 (15)	0.0458 (16)	0.0547 (19)	0.0022 (13)	0.0062 (15)	−0.0038 (13)

### Geometric parameters (Å, °)

**Table:**

O1—C7	1.326 (3)	C2—C9	1.494 (4)
O1—C8	1.454 (4)	C3—C4	1.375 (4)
O2—C7	1.191 (3)	C4—C5	1.368 (4)
O3—N1	1.218 (4)	C5—C6	1.369 (4)
O4—N1	1.214 (3)	C4—H4	0.9300
O5—N2	1.204 (4)	C6—H6	0.9300
O6—N2	1.212 (4)	C8—H8A	0.9600
N1—C3	1.482 (4)	C8—H8B	0.9600
N2—C5	1.474 (4)	C8—H8C	0.9600
C1—C2	1.405 (4)	C9—H9A	0.9600
C1—C6	1.388 (4)	C9—H9B	0.9600
C1—C7	1.493 (4)	C9—H9C	0.9600
C2—C3	1.395 (3)		
			
C7—O1—C8	116.3 (2)	C1—C6—C5	120.0 (2)
O3—N1—O4	124.7 (3)	O1—C7—O2	123.1 (3)
O3—N1—C3	118.4 (3)	O1—C7—C1	111.4 (2)
O4—N1—C3	116.9 (2)	O2—C7—C1	125.5 (2)
O5—N2—O6	123.9 (3)	C3—C4—H4	122.00
O5—N2—C5	118.4 (3)	C5—C4—H4	122.00
O6—N2—C5	117.7 (2)	C1—C6—H6	120.00
C2—C1—C6	120.9 (2)	C5—C6—H6	120.00
C2—C1—C7	121.4 (2)	O1—C8—H8A	109.00
C6—C1—C7	117.7 (2)	O1—C8—H8B	109.00
C1—C2—C3	115.1 (2)	O1—C8—H8C	109.00
C1—C2—C9	123.0 (2)	H8A—C8—H8B	109.00
C3—C2—C9	121.8 (2)	H8A—C8—H8C	109.00
N1—C3—C2	118.8 (2)	H8B—C8—H8C	110.00
N1—C3—C4	115.8 (2)	C2—C9—H9A	109.00
C2—C3—C4	125.4 (2)	C2—C9—H9B	109.00
C3—C4—C5	116.5 (2)	C2—C9—H9C	109.00
N2—C5—C4	118.6 (2)	H9A—C9—H9B	109.00
N2—C5—C6	119.2 (2)	H9A—C9—H9C	110.00
C4—C5—C6	122.2 (3)	H9B—C9—H9C	109.00
			
C8—O1—C7—O2	−0.4 (5)	C7—C1—C6—C5	−178.3 (2)
C8—O1—C7—C1	178.9 (3)	C2—C1—C7—O1	157.0 (2)
O3—N1—C3—C2	60.3 (3)	C2—C1—C7—O2	−23.7 (4)
O3—N1—C3—C4	−121.3 (3)	C6—C1—C7—O1	−24.1 (3)
O4—N1—C3—C2	−119.0 (3)	C6—C1—C7—O2	155.2 (3)
O4—N1—C3—C4	59.4 (3)	C1—C2—C3—N1	176.8 (2)
O5—N2—C5—C4	4.3 (4)	C1—C2—C3—C4	−1.4 (4)
O5—N2—C5—C6	−176.2 (3)	C9—C2—C3—N1	0.4 (4)
O6—N2—C5—C4	−175.8 (3)	C9—C2—C3—C4	−177.8 (3)
O6—N2—C5—C6	3.7 (4)	N1—C3—C4—C5	−178.2 (2)
C6—C1—C2—C3	1.1 (3)	C2—C3—C4—C5	0.1 (4)
C6—C1—C2—C9	177.4 (2)	C3—C4—C5—N2	−178.9 (2)
C7—C1—C2—C3	179.9 (2)	C3—C4—C5—C6	1.7 (4)
C7—C1—C2—C9	−3.8 (4)	N2—C5—C6—C1	178.5 (2)
C2—C1—C6—C5	0.6 (4)	C4—C5—C6—C1	−2.0 (4)

### Hydrogen-bond geometry (Å, °)

**Table:**

*D*—H···*A*	*D*—H	H···*A*	*D*···*A*	*D*—H···*A*
C9—H9C···O2^i^	0.96	2.56	3.353 (4)	140

Symmetry codes: (i) *x*−1/2, −*y*+1/2, −*z*.
